# Lipidomic Analysis of Extracellular Vesicles from the Pathogenic Phase of *Paracoccidioides brasiliensis*

**DOI:** 10.1371/journal.pone.0039463

**Published:** 2012-06-22

**Authors:** Milene C. Vallejo, Ernesto S. Nakayasu, Larissa V. G. Longo, Luciane Ganiko, Felipe G. Lopes, Alisson L. Matsuo, Igor C. Almeida, Rosana Puccia

**Affiliations:** 1 Departamento de Microbiologia, Imunologia e Parasitologia, Universidade Federal de São Paulo, UNIFESP, São Paulo, São Paulo, Brazil; 2 Border Biomedical Research Center, Department of Biological Sciences, University of Texas at El Paso (UTEP), El Paso, Texas, United States of America; Montana State University, United States of America

## Abstract

**Background:**

Fungal extracellular vesicles are able to cross the cell wall and transport molecules that help in nutrient acquisition, cell defense, and modulation of the host defense machinery.

**Methodology/Principal Findings:**

*Here we present a detailed lipidomic analysis of extracellular vesicles released by Paracoccidioides brasiliensis at the yeast pathogenic phase. We compared data of two representative isolates, Pb3 and Pb18, which have distinct virulence profiles and phylogenetic background. Vesicle lipids were fractionated into different classes and analyzed by either electrospray ionization- or gas chromatography-mass spectrometry. We found two species of monohexosylceramide and 33 phospholipid species, including phosphatidylcholine, phosphatidylethanolamine, phosphatidic acid, phosphatidylserine, phosphatidylinositol, and phosphatidylglycerol. Among the phospholipid-bound fatty acids in extracellular vesicles, C18*1 predominated in Pb3, whereas C18:2 prevailed in Pb18. The prevalent sterol in Pb3 and Pb18 vesicles was brassicasterol, followed by ergosterol and lanosterol. Inter-isolate differences in sterol composition were observed, and also between extracellular vesicles and whole cells.

**Conclusions/Significance:**

The extensive lipidomic analysis of extracellular vesicles from two *P. brasiliensis* isolates will help to understand the composition of these fungal components/organelles and will hopefully be useful to study their biogenesis and role in host-pathogen interactions.

## Introduction

Fungal extracellular vesicles are able to concentrate and transport outward from the cell wall large molecular weight components that can potentially interfere with the host’s barriers and immunodefense, such as enzymes, adhesins, and antioxidant proteins [1]. Released fungal vesicles have originally been characterized in the opportunistic fungus *Cryptococcus neoformans* [2], where important virulence factors such as capsular glucuronoxylomannan (GXM), the pigment melanin, phosphatases, urease, and laccase were found [2]–[4]. *C. neoformans* vesicles are internalized by macrophages *in vitro*, inducing phagocytosis, microbicidal activity, and the production of tumor necrosis factor α (TNF-α), interleukin-10 (IL-10), and transforming growth factor β (TGF-β) [5]. Therefore, fungal extracellular vesicles may represent a general mechanism of host immune-modulation, as also recently suggested for *Malassezia sympodialis* [6]. By transmission electron microscopy, extracellular vesicle populations with diverse size and electron densities were detected in *C. neoformans* [2], [3], *Histoplasma capsulatum*, *Candida albicans*, *C. parapsilosis*, *Sporothrix schenckii* [7], and *Saccharomyces cerevisiae* [8]. This morphological heterogeneity pointed to the existence of multiple mechanisms of vesicle biogenesis, as recently suggested [1], [8]–[10].

We have recently characterized extracellular vesicles in the thermal dimorphic pathogen *Paracoccidioides brasiliensis* [11], which causes paracoccidioidomycosis (PCM). This is a systemic mycosis prevalent in Latin America, where it is estimated that 10 million individuals are infected [12]. PCM infection starts by inhalation of environmental fungal conidia, which develop as multibudding yeasts in pulmonary alveoli [12]. Active PCM affects mainly the lungs, but dissemination to other sites is frequent. Modulation of the immune system determines the fate of infection and the Th1 branch of cellular immunity is the main source of host protection [13]. Therefore, fungal components that elicit immune response and/or promote other types of interaction with the host will shape the infection features. Morphological changes in *P. brasiliensis* are followed by modifications in the cell wall polysaccharide composition [12], which are accompanied by migration and reorganization of membrane lipids, mainly glycosphingolipids (GSLs) [14], [15]. These cell wall and lipid membrane components are often associated with fungal pathogenesis [16]–[18].

We have shown that *P. brasiliensis* extracellular vesicles carry antigenic molecules that are recognized by sera from patients with PCM; among them are α-linked galactopyranosyl (α-Gal) epitopes that are partially contained in protein *O*-linked oligosaccharides [11]. We have also compared fungal extracellular vesicle proteomes from *P. brasiliensis, C. neoformans* [3], *H. capsulatum* [7], and *S. cerevisiae* [8] and found a total of 72 (35%) *P. brasiliensis* vesicular proteins with orthologues reported in at least two other fungal species [19].

There is only scarce information about fungal extracellular vesicle lipid composition [2], [7], [20], while there are few and more complete lipidomic studies of mammalian cell exosomes [21]–[25]. In a detailed analysis, B-cell exosomes were enriched in cholesterol, sphingomyelin, and ganglioside GM3, which are typically found in detergent-resistant membrane domains [26]. Thus, these detergent-resistant membrane domains could play an important role in the generation of membrane buds and even in membrane fission during exosome formation.

Previous literature data showed that particles coated with total lipids or enriched fractions of fatty acid and triglycerides extracted from *P. brasiliensis* induced an intense granulomatous reaction in mice [27], [28], suggesting that lipids extracted from *P. brasiliensis* are able to induce inflammatory reactions. Here we have performed extensive lipidomic analysis of extracellular vesicles from isolates Pb3 and Pb18. Pb18 is a member of the major paraphyletic S1 group [29] and is extensively studied due to its high virulence [13]; Pb3 belongs to a cryptic PS2 group [29], whose members evoked regressive infection when compared with S1 samples [30]. Presently, we have detected differences in extracellular vesicle lipid composition that might potentially account for these distinct disease outcomes.

## Materials and Methods

### Lipid Extraction and Fractionation

*P. brasiliensis* isolates Pb3 and Pb18 were cultivated in 500 mL Ham’s F-12 medium (Invitrogen) supplemented with 1.5% glucose, at 36°C (yeast pathogenic phase) with shaking for 2 days, starting from cell pellets of four four-day-old pre-inoculums. Extracellular vesicles were isolated from cell-free culture supernatants (one preparation  = 500 mL culture) as previously described [11]. Lipidomic analysis was carried out using the full contents of 2.5 preparations of each fungal isolate. Yeast cells and extracellular vesicle lipids from Pb3 and Pb18 were sequentially extracted (three times each) with 1 mL of chloroform:methanol (2∶1, v:v) and chloroform:methanol:water (1∶2:0.8, v:v:v). Organic extracts were combined, dried under N_2_ stream, and dissolved in 2 mL chloroform. Lipids were then fractionated in a silica-60 column prepared in a Pasteur pipette using a very fine wool glass at the tip and approximately 500 mg of silica-gel 60 spheres (pore size 60 Å, 200–400 mm mesh, Sigma-Aldrich, St. Louis, MO). The column was washed sequentially with 5 mL each of methanol, acetone, and chloroform. Extracted lipids were loaded and sterols were eluted with chloroform (5 mL), followed by elution of glycolipids with acetone (5 mL), and then phospholipids with methanol (5 mL). Collected lipid fractions were dried under N_2_ stream.

### Permethylation of Glycolipids

Glycolipids were permethylated according to Ciucanu and Kerek [31]. Dried glycolipid fractions were stored in a container with desiccant silica at −20°C, overnight, to remove the moisture. Samples were resuspended in 150 µL of dimethyl sulfoxide (DMSO) (Sigma-Aldrich), vortexed vigorously with a few milligrams of powdered NaOH (Sigma-Aldrich), then mixed with 80 µL of iodomethane (Sigma-Aldrich), and incubated for 1 h at room temperature under shaking. The reaction mixture was quenched with 2 mL of water and 2 mL of dichloromethane (DCM), centrifuged to remove the aqueous phase (top layer), and the organic phase was washed three times with 2 mL of water. The final organic phase was collected into a fresh tube, dried under N_2_ stream, and suspended in HPLC-grade methanol (CHROMASOLV, Sigma-Aldrich) for mass spectrometry analysis.

### Methylation of Fatty Acids

Methylation of fatty acids was performed according to Maldonado et al. [32]. The phospholipid fraction was resuspended in 400 µL 13 N ammonium hydroxide:methanol (1∶1, v:v) and incubated for 1 h at 37°C. Samples were then dried under N_2_ stream, followed by two cycles of dissolving in anhydrous methanol and completely drying to remove remaining ammonium hydroxide. Methylation was carried out by incubating the samples in 400 µL 0.5 N methanolic HCl (Supelco, Sigma-Aldrich) for 1 h at 75°C followed by neutralization with 400 µL 0.5 N NaOH. To remove salts from the reaction, samples were partitioned with 1.5 mL water and 1.5 mL DCM. After two additional washes in water, the organic phase composed of fatty acids was separated and concentrated under N_2_ stream to a final volume of ∼100 µL.

### Analysis of Phospholipids and Glycolipids

Glycolipids and phospholipids were analyzed by electrospray ionization-tandem mass spectrometry (ESI-MS/MS) on a linear ion-trap mass spectrometer (LTQ XL, ThermoFisher Scientific, San Jose, CA) coupled with an automated nanoinfusion/nanospray source (Triversa NanoMate System, Advion). Phospholipid samples were analyzed in negative-ion mode, dissolved in methanol containing 0.05% formic acid (FA) and 0.05% NH_4_OH or in the positive-ion mode in methanol with 10 mM LiOH. Full-scan spectra were collected at the 500–1000 *m/z* range, and samples were subjected to total-ion mapping (TIM) (2 a.m.u. of isolation width; PQD fragmentation to 29% normalized collision energy; activation Q of 0.7; and activation time of 0.1 ms). Permethylated glycolipids were analyzed in the positive-ion mode, as described above for phospholipids, but they were dissolved in pure methanol and full-scan spectra were collected at the 500–2000 *m/z* range. MS/MS spectra were analyzed manually for identification of lipid species as described in detail elsewhere [33].

### Analysis of Sterols and Fatty Acids

Sterols were suspended in DCM and analyzed by gas chromatography-mass spectrometry (GC-MS, Trace GC Polaris Q, Thermo Fisher Scientific) using a TR5-MS column (30 m×250 µm×0.25 µm, Thermo Fisher Scientific). The injector temperature was set to 250°C and the gradient was held for 3 min at 170°C, followed by an increment of 20°C/min up to 280°C, which was then maintained for 17 min. Helium was used as a carrier gas at a flow rate of 1.2 mL/min. The molecules were ionized in the positive-ion mode by electron impact at 70 eV and 200°C. Spectra analysis was carried out by searching the spectral library and by comparison with external standards of brassicasterol, ergosterol and lanosterol (Avanti Polar Lipids, Inc., Alabaster, AL), and cholesterol (Sigma-Aldrich).

Methylated fatty acid samples were concentrated to ∼100 µL of DCM and 1 µL was used for analysis in GC-MS. Samples were separated in a SP-2380 fused silica column (30 m×250 µm×0.20 µm, Supelco). The injector was set at 200°C, and the following temperature gradient was used: 70°C for 5 min, followed by an increment of 4°C/min up to 140°C, 2°C/min up to 185°C, and 185°C for 10 min. Helium was used as carrier gas at 1 mL/min flow rate. The molecules were ionized by electron impact in the positive-ion mode with 70 eV and 200°C. The spectra were collected at the 30–400 *m/z* range, and extracted ion chromatograms were generated by plotting the diagnostic fragment-ion species at *m/z* 41, 43, and 55. Fatty acid species were identified by comparison with a FAME 37 mix external standard (Supelco).

## Results

### 1. ESI-MS/MS Analysis of Phospholipids

**Figure 1:**
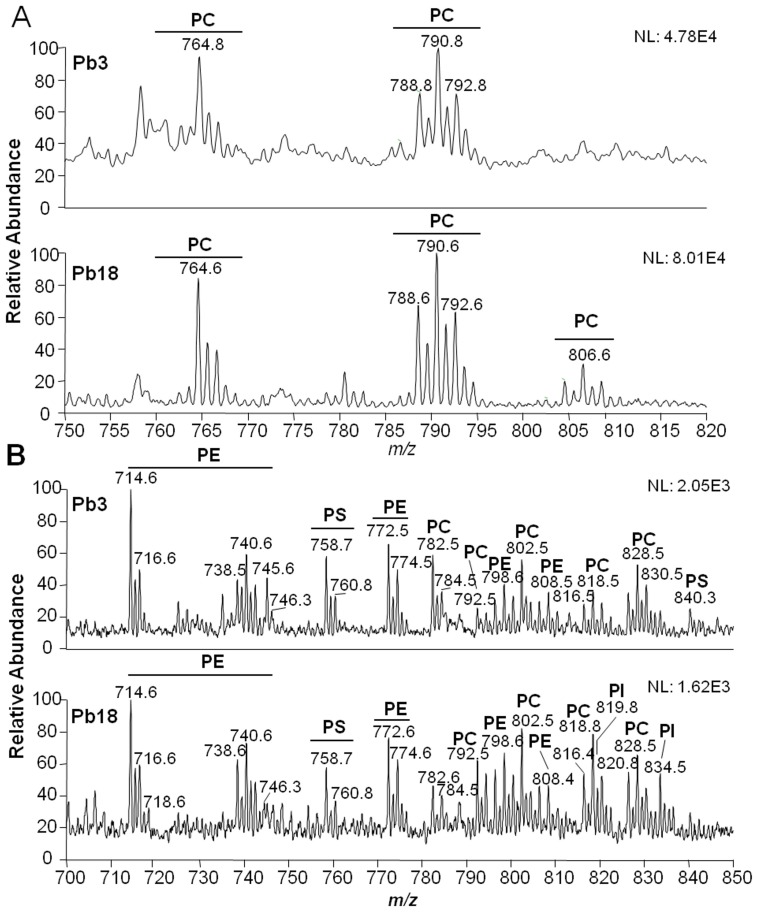
Phospholipid analysis of Pb3 and Pb18 extracellular vesicles by ESI-MS/MS. Phospholipid-enriched fractions from total extracellular vesicle lipids were analyzed by ESI-MS in the (A) positive- and (B) negative-ion modes, followed by total-ion mapping (TIM) fragmentation. The ion species corresponding to the identified phospholipids are indicated as follows: phosphatidylethanolamine (PE), phosphatidylcholine (PC), phosphatidylserine (PS), and phosphatidylinositol (PI). *m/z*, mass to charge ratio.

Given that phospholipids are major components of cellular membrane bilayers and probably of extracellular vesicles, we performed a detailed characterization of phospholipids from vesicle membranes. Total vesicle lipids were extracted with organic solvents, fractionated in a silica-60 column, and analyzed by ESI-MS/MS in the positive- and negative-ion modes. In the positive-ion mode, only phosphatidylcholine (PC) species (four in total) were identified (Fig. 1A, Table 1). They included PC species bearing different adducts (data not shown in Table 1), such as *m/z* 790.6 ([M – H + Li]^+^, C18:2/C18:1-PC) (Fig. 1A). An MS/MS spectrum of a major PC species is shown in Fig. S1. The diagnostic fragment ion for PC species is a neutral loss of 59 Da corresponding to the trimethylamine ((CH_3_)_3_N or Me_3_N) of the head group from sodiated or lithiated species ([M – Me_3_N + Li (or Na)]^+^). Fatty acid chains can also be identified by neutral-loss fragments ([M – fatty acid + Li (or Na)]^+^) or double losses of fatty acid and Me_3_N groups ([M – fatty acid – Me_3_N + Li (or Na)]^+^) [33].

**Table 1:** Composition of major phospholipids identified by ESI-MS total-ion mapping from Pb3 and Pb18 vesicles.

	Observed species							
	Pb3		Pb18				Normalized intensity	
Ion-mode	Ion species	*m/z*	Ion species	*m/z*	ProposedComposition	Predicted Mass (Da)	Pb3	Pb18
**Phosphatidylethanolamine (PE; n = 13)**								
(−)	[M - H]^−^	476.6	[M - H]^−^	476.5	*lyso*-C18:2	477.3	TR	TR
(−)	[M - H]^−^	662.5	ND	ND	C14:0/C16:0	663.5	TR	ND
(−)	[M - H]^−^	686.6	[M - H]^−^	686.7	C14:0/C18:2	687.4	0.2	0.4
(−)	[M - H]^−^	700.7	[M - H]^−^	700.6	C15:0/C18:2	701.5	TR*	TR
(−)	[M - H]^−^	702.7	[M - H]^−^	702.7	C15:0/C18:1	703.5	TR	TR
(−)	[M - H]^−^	714.7	[M - H]^−^	714.7	C16:0/C18:2	715.5	1.0	1.0
(−)	[M - H]^−^	716.7	[M - H]^−^	716.7	C16:0/C18:1	717.5	0.5	0.6
(−)	[M - H]^−^	738.8	[M - H]^−^	738.7	C18:2/C18:2	739.5	0.3	0.4
(−)	[M - H]^−^	740.7	[M - H]^−^	740.7	C18:2/C18:1	741.5	0.6	0.8
(−)	[M - H]^−^	742.7	ND	ND	C18:1/C18:1	743.5	0.3	ND
(−)	[M - H]^−^	744.7	[M - H]^−^	744.8	C18:0/C18:1	745.6	0.4	0.5
(−)	[M - H]^−^	746.7	[M - H]^−^	746.7	C16:0/C20:0	747.6	0.4	0.5
(−)	ND	ND	[M - H]^−^	746.7	C18:0/C18:0	747.6	ND	0.5
**Phosphatidylcholine (PC; n = 12)**								
(−)	[M - H + Cl]^−^	554.5	ND	ND	*lyso*-C18:2	520.3	TR	ND
(−)	[M - H + HCOO]^−^	566.5	ND	ND	*lyso*-C18:3	522.3	TR	ND
(−)	[M - H + HCOO]^−^	788.6	ND	ND	C15:0/C18:2	744.6	0.2	ND
(−)	[M - H + Cl]^−^	792.7	[M - H + Cl]^−^	792.8	C16:0/C18:2	758.6	0.3	0.5
(+)	[M - H + Li]^−^	764.8	[M - H + Li]^−^	764.6	C16:0/C18:2	758.6	TR	TR
(−)	[M - H + HCOO]^−^	804.7	[M - H + HCOO]^−^	804.7	C16:0/C18:1	760.6	0.3	0.3
(−)	[M - H + Cl]^−^	816.7	[M - H + HCOO]^−^	826.6	C18:2/C18:2	782.6	0.3	0.5
(+)	[M - H + Li]^−^	788.8	[M - H + Li]^−^	788.6	C18:2/C18:2	782.6	TR	TR
(−)	[M - H + Cl]^−^	818.8	ND	ND	C18:2/C18:1	784.6	0.3	ND
(+)	[M - H + Li]^−^	790.7	[M - H + Li]^−^	790.6	C18:2/C18:1	784.6	TR	ND
(−)	[M - H + HCOO]^−^	830.7	ND	ND	C18:1/C18:1	786.6	0.4	ND
(+)	[M - H + HCOO]^−^	830.7	[M - H + Li]^−^	792.7	C18:1/C18:1	786.6	TR	TR
**Phosphatidic acid (PA; n = 6)**								
(−)	ND	ND	[M - H]^−^	657.8	C15:0/C18:2	658.5	.	TR
(−)	[M - H]^−^	671.6	ND	ND	C16:0/C18:2	672.5	TR	TR
(−)	[M - H]^−^	673.6	[M - H]^−^	673.6	C16:0/C18:1	674.5	TR	TR
(−)	[M - H]^−^	695.6	[M - H]^−^	695.6	C18:2/C18:2	696.5	TR	TR
(−)	[M - H]^−^	697.7	[M - H]^−^	697.6	C18:2/C18:1	698.5	TR	0.2
(−)	[M - H]^−^	699.7	[M - H]^−^	699.7	C18:1/C18:1	701.0	TR	TR
**Phosphatidylserine (PS; n = 2)**								
(−)	[M - H]^−^	758.7	[M - H]^−^	758.7	C16:0/C18:2	759.5	0.3	0.2
(−)	[M - H]^−^	760.6	[M - H]^−^	760.8	C16:0/C18:1	761.5	0.3	0.3
**Phosphatidylglycerol (PG; n = 1)**								
(−)	[M - H]^−^	747.6	[M - H]^−^	747.8	C16:0/C18:1	748.5	0.4	0.3
**Phosphatidylinositol (PI; n = 3)**								
(−)	ND	ND	[M - H]^−^	819.8	Alkyl-C16:0-Acyl-C18:2	820.5	ND	0.5
(−)	ND	ND	[M - H]^−^	821.8	Alkyl-C16:0-Acyl-C18:1	822.5	ND	0.5
(−)	[M - H]^−^	833.7	[M - H]^−^	833.7	C16:0/C18:2	834.5	TR	0.5

a ND, not detected.

b TR, trace amounts.

c Normalized by the intesity of PE-C16:0/C18:2 at m/z 714.7.

The major diagnostic ion for phospholipids in the negative-ion mode is *m/z* 153, corresponding to dehydrated glycerophosphate [GroP – H_2_O – H]^–^. Further specific diagnostic ions were used here to identify each class of phospholipids. For instance, phosphatidylethanolamine (PE) species were identified by the fragment ion at *m/z* 196, corresponding to dehydrated glycerophosphoethanolamine [GroPEtNP – H_2_O]^−^ (Fig. S2). Tandem-MS analysis of chlorinated and formiated PC species in the negative-ion mode led to the neutral losses of 50 or 60 Da, corresponding to the loss of the N-methyl group of quaternary nitrogen and the chloride adduct as methylchloride [M – MeCl – H]^−^, or the N-methyl group and formate adduct as methylformate [M – HCOOMe – H]^−^ (Fig. S3). The neutral loss of 87 Da [M – Ser – H]^−^ was used for identification of phosphatidylserine (PS) species (Fig. S4). The fragment at *m/z* 241, corresponding to dehydrated phosphoinositol [InsP – H_2_O – H]^−^, was used for the identification of phosphatidylinositol (PI) species (Fig. S5) [33]. Unfortunately, the fragmentation of phosphatidylglycerol (PG) (Fig. S6) and phosphatidic acid (PA) head groups (Fig. S7) also resulted in dehydrated glycerophosphate fragment ion; however, they could be distinguished by the molecular mass of the intact parent ion. Since fatty acids ionize in the negative-ion mode as carboxylate ions, they could easily be identified after phospholipid fragmentation, although the unsaturation position or the *cis*/*trans* configuration could not be determined by this type of analysis. In total, the negative-ion mode analysis led to the identification of 33 phospholipid species, being 8 PC, 13 PE, 6 PA, 2 PS, 3 PI, and one PG, with distinct fatty acid chains (Fig. 1B, Table 1).

We next compared Pb3 and Pb18 extracellular vesicle phospholipid profiles in negative-ion ESI-MS by normalizing the signal intensity of each phospholipid species by the most intense ion at *m/z* 714.6 (Fig. 1B), which corresponds to C16:0/C18:2-PE (Table 1). In general, similar phospholipid profiles were observed in both positive- and negative-ion modes (Fig. 1). The major difference between isolates was the presence of two abundant species of PI in Pb18, specifically, alkyl-C16:0-acyl-C18:1 (*m/z* 821.8) and alkyl-C16:0-acyl-C18:2 (*m/z* 819.8), which were not detected in Pb3 extracellular vesicles (Table 1) by TIM analysis (data not shown).

### 2. GC/MS Analysis of Fatty Acids

**Figure 2:**
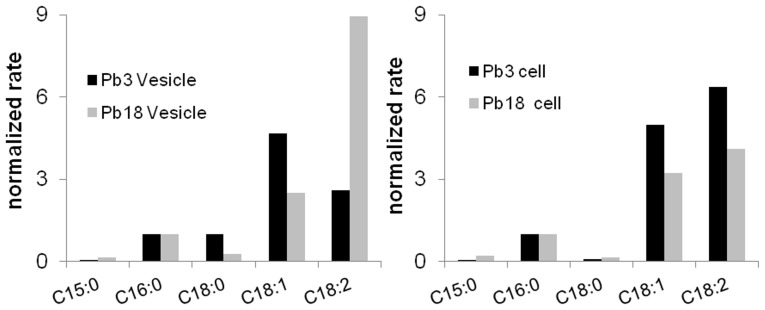
Phospholipid fatty acid analysis of Pb3 and Pb18 extracellular vesicles and whole yeast cells. Fatty acids from phospholipid-enriched fractions resulting from silica gel-60 column separation were released with NH_4_OH, methylated with methanolic HCl, and analyzed by GC-MS. Signal intensity was normalized by the C16:0 peak area.

In order to complement the vesicle phospholipid characterization by ESI-MS/MS, we performed a fatty acid compositional analysis. Fatty acids were extracted from phospholipids, methylated, and analyzed by GC-MS. Different species were identified by comparison with an external standard and the results showed differences between Pb3 and Pb18 (Fig. 2). In our analysis we could not reliably determine the unsaturation position. However, they were all identified to have double bonds in the *cis* configuration. The most frequently found monounsaturated fatty acids have their double bound between C9 and C10 (Δ^9^), whereas double unsaturation happens generally at positions Δ^12^ and Δ^15^ [34]. Therefore, fatty acid C18:1 identified in our analysis is possibly *cis*-9-octadecenoic acid, or oleic acid, while C18:2 is probably *cis*-,*cis*-9,12-octadecadienoic, also known as linoleic acid. The predominant fatty acid in Pb3 extracellular vesicles was C18:1, followed by C18:2. Stearic acid (C18:0) was detected in similar abundance as palmitic acid (C16:0). In contrast, the most abundant fatty acid in Pb18 extracellular vesicle phospholipids was C18:2, followed by C18:1, palmitic, and stearic acid. Traces of pentadecanoic acid (C15:0) were also identified in both extracellular vesicle preparations (Fig. 2). Interestingly, composition analysis of whole yeast cells revealed C18:2 as predominant in both Pb3 and Pb18 cells, followed closely by C18:1. In Pb18 samples, the amount of C18:2 was higher in both vesicles and cells, but the ratio between C18:2/C18:1 in Pb18 vesicles was 3.6, whereas in cells it was only 1.3. Palmitic acid was detected less abundantly, but equally well in both isolates. Taken together, our data showed differences in fatty acid composition between extracellular vesicles from distinct isolates, and also between extracellular vesicles and whole yeasts.

### 3. GC/MS Analysis of Sterols

**Figure 3:**
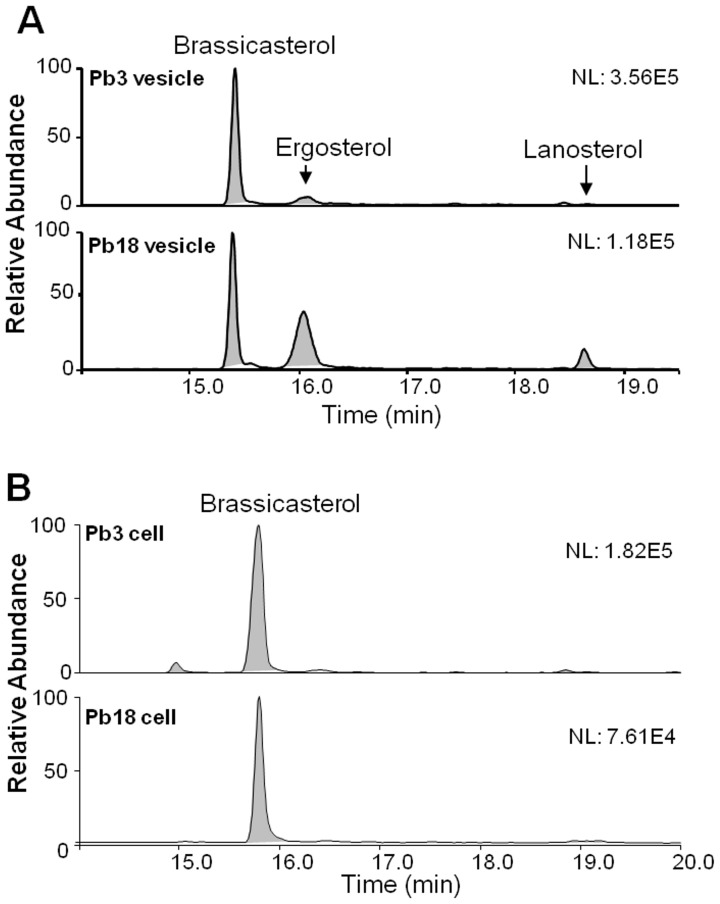
GC-MS sterol analysis of Pb3 and Pb18 extracellular vesicles. Sterol-enriched fractions of total lipids extracted from Pb3 and Pb18 extracellular vesicles (A) or yeast cells (B) were directly analyzed by GC-MS without previous derivatization. Extracted-ion chromatograms were generated by plotting diagnostic fragment ions for sterol species at *m/z* 368, 386, 384, 398, 395, 396, 412, and 426.

Sterol fractions from silica-gel 60 column were analyzed by GC-MS, and species were identified by both using external standards and searching the spectral library. Sterol species identified in both Pb3 and Pb18 extracellular vesicles were brassicasterol (ergosta-5,22-dien-3β-ol), ergosterol (ergosta-5,7-22-trienol), and lanosterol (lanosta-8,24-dien-3-ol) (Fig. 3A). In order to compare the relative abundance of sterols in Pb3 and Pb18 extracellular vesicles, we calculated the peak area ratios between brassicasterol and ergosterol, and brassicasterol and lanosterol. The results showed that brassicasterol predominated in Pb3 with ratios of 7.9 (brassicasterol/ergosterol) and 94.5 (brassicasterol/lanosterol). In Pb18, brassicasterol and ergosterol relative abundances were comparative (ratio of 1.13), while brassicasterol predominated over lanosterol at a lower ratio (5.4) (Table 2). Therefore, we found comparatively more ergosterol and lanosterol in Pb18 than Pb3 vesicles.

**Table 2:** Composition of Pb3 and Pb18 extracellular vesicle and whole yeast cell total sterols identified by GC-MS. TR, trace amounts.

	peak areas			ratio	
*P. brasiliensis*	brassicasterol	ergosterol	lanosterol	bras/erg	bras/lan
**Pb3 vesicles**	1624766	205663	17200	7.9	94.5
**Pb18 vesicles**	522066	459758	96336	1.13	5.4
**Pb3 cells**	1411649	TR	TR	.	.
**Pb18 cells**	474239	TR	TR	.	.

In order to compare extracellular vesicle and yeast cell sterols, we extracted total lipids from Pb3 and Pb18 whole cells and analyzed the sterol fraction by GC-MS. Brassicasterol was the single major sterol detected, while trace amounts of ergosterol and lanosterol were also seen (Fig. 3B and Table 2).

### 4. ESI-MS/MS Analysis of Neutral Glycolipids

**Figure 4:**
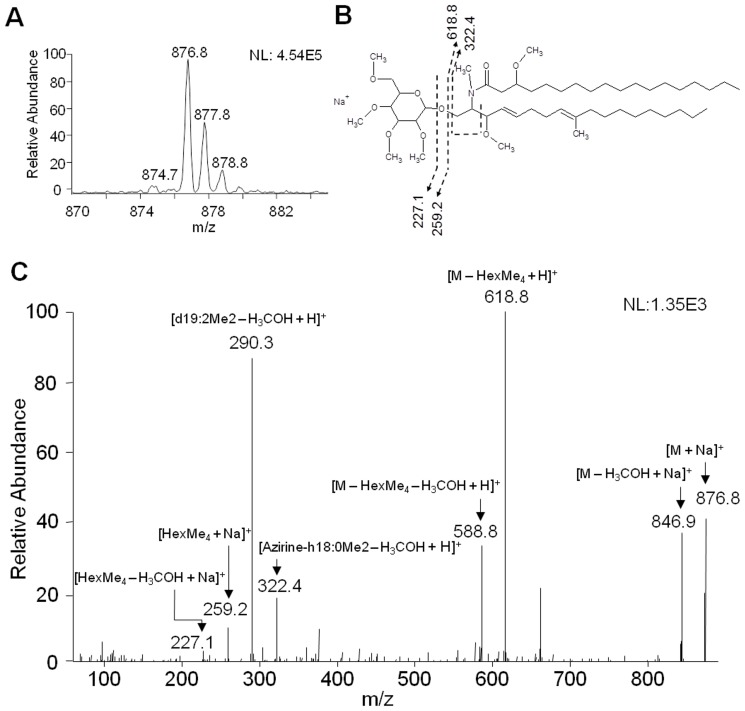
Neutral glycolipid analysis of Pb3 and Pb18 extracellular vesicles. Glycolipid-enriched fractions from total lipids were permethylated and analyzed by ESI-MS/MS. (A) Full-scan spectrum by ESI-MS in the positive-ion mode of Pb3 extracellular vesicle glycolipids. (B) Proposed fragmentation and structure of the major glycolipid identified in Pb3 and Pb18 extracellular vesicles. The positions of unsaturation, hydroxyl and methyl groups of the ceramide were based on the structure proposed by Toledo et al. (1999). (C) MS/MS spectrum of the major glycolipid species at *m/z* 876.8. The number at the top right corner indicates ion intensity. *m/z*, mass to charge ratio. The same glycolipid species have been identified in Pb3 and Pb18, however since the analysis is qualitative we cannot assure that are not quantitative differences.

Glycosphingolipids (GSLs) are ceramide-containing lipids covalently attached to mono- or oligosaccharide core. Total lipids from extracellular vesicles were separated in a silica-gel 60 column, and the fractions enriched in neutral glycolipids were permethylated and examined by positive-ion mode ESI-MS/MS. Full scan from 500 to 2000 *m/z* range revealed a major singly charged ion species at *m/z* 876.8 and a minor one at *m/z* 874.7 in both Pb3 and Pb18 samples (Fig. 4A). Both ion species (*m/z* 876.8 and 874.7) were subjected to fragmentation and the resulting spectra analyzed manually. Tandem-MS spectrum of the parent ion at *m/z* 876.8 showed diagnostic ions for glycolipids corresponding to methylated hexose at *m/z* 227.1 [HexMe_4_– H_3_COH + Na]^+^ and 259.2 [HexMe_4_+ Na]^+^, in addition to their corresponding neutral loss fragments at *m/z* 588.8 [M – HexMe_4_– H_3_COH + Na]^+^ and 618.8 [M – HexMe_4_+ Na]^+^ (Fig. 4B, C). The fragment ion at *m/z* 290.3 corresponds to the mass of a permethylated d19:2 sphingoid base [d19:2Me_2_– H_3_COH + H]^+^ (Fig. 4B, C). The fragment ion at *m/z* 322.4 corresponds to a fatty acid attached to part of the sphingoid base [Azirine-h18:0Me_2_– H_3_COH + H]^+^ (Fig. 4B, C). Thus, the most probable structure corresponding to the abundant peak *m/z* 876.8 is Hex-C18:0-OH/d19:2-Cer. When subjected to tandem MS/MS analysis, the minor neutral glycolipid species observed at *m/z* 874.7 had similar structure, except for an unsaturation in the fatty acid residue Hex-C18:1-OH/d19:2-Cer (data not shown).

The type of ESI-MS/MS analysis carried out here did not enable us to define the position of the double bond, and hydroxyl and methyl groups in the sphingoid base. However, Toledo et al. [14] analyzed glycosphingolipids of Pb18 cells by nuclear magnetic resonance (NMR) and identified two species consistent with our findings. Based on their analysis, the sphingoid base is most probably methylated at C9 and the double bonds are in the *cis* configuration at C4 and C8. That suggests that the neutral glycosphingolipid peaks at *m/z* 876.8 and 874.7 correspond, respectively, to *N*-2′-hydroxyoctadecanoate (4*E*,8*E*)-9-methyl-4,8-sphingadienine, and to *N*-2′-hydroxy-(*E*)-3′-octadecenoate (4*E*,8*E*)-9-methyl-4,8-sphingadienine.

## Discussion

Here we report the lipidomic analysis of extracellular vesicle preparations present in cell-free culture supernatants from the pathogenic yeast phase of *P. brasiliensis* cultivated in defined medium supplemented with glucose. We performed the most detailed lipidomic analysis possible, considering the scarce amounts of material contained in these preparations, whose yields are generally limited. Nonetheless, we have been able to identify 33 species of phospholipids, besides fatty acids, neutral glycosphingolipids and sterols. We believe that we are contributing to this type of structural analysis with significant results considering the restricted data so far available for fungal extracellular vesicle lipids [2], [7], [20].

An interesting and original aspect of our work was the qualitative data comparison between Pb18 and Pb3 isolates, which represent two distant *P. brasiliensis* phylogenetic groups that seem to evoke distinct disease outcomes in mice [29], [30]. In the B10.A mice model, Pb3 evoked milder infection than Pb18 [30]. The antibody responses elicited by Pb3 were richer in IgG2a, IgG2b, and IgG3, suggesting a predominant Th1 type of host immunity, as opposed to predominant IgG1 and IgA detected in Pb18-infected mice. Accordingly, mice infected with Pb3 secreted higher amounts of protective IFN-γ in the lungs and no IL-10, which was only detected in Pb18-infected mice (Carvalho and Puccia, unpublished). In addition, we have observed that TNF-α production was higher when macrophages were stimulated with vesicles isolated from Pb3, whereas IL-10 expression was higher when macrophages were stimulated with Pb18 vesicles (Vallejo et al., unpublished). Differential aspects of extracellular vesicle lipid composition, as detected in our work, could be at least partially responsible for these results, but that remains to be experimentally proven. Low yields of lipidic fractions have so far hampered our attempts to address this question using isolated extracellular vesicle lipids.

In both Pb3 and Pb18 extracellular vesicles, the main classes of phospholipids were represented, but the number of species varied in each class. Phosphatidylcholine, phosphatidylethanolamine, phosphatidic acid, phosphatidylserine, phosphatidylglycerol and phosphatidylinositol, presently detected, have previously been described in total cell extracts from different *P. brasiliensis* isolates [35], [36]. In *H. capsulatum* extracellular vesicles, 17 species were described, being six PE, three PS, and eight PC species, which were similar to those found in total cell extracts [7]. In *C. neoformans* vesicles PC was identified, which is also the major phospholipid found in this fungal membrane [20], [37]. We detected a total of 33 species in Pb3 and Pb18, and although the profiles were generally similar for both isolates, C18:0/C:18:0-PE, and two peaks of alkyl-C16:0-acyl-C18:2 or –C18:1-PI were only seen in Pb18. Species detected exclusively in Pb3 were C15:0/C:18:2-, C18:2/C18:1-, and C18:1/C18:1-PC, besides C18:1/C18:1-PE. The presence of alkyl-C16:0-acyl-C18:2 or –C18:1-PI was particularly interesting since the alkylacylglycerol unsaturated fatty acid present in glycosylphosphatidylinositol from the trypomastigote form of the parasite *Trypanosoma cruzi* is essential to induce cytokine production [38].

Fatty acid analysis of phospholipids showed intrinsic differences between Pb3 and Pb18 extracellular vesicles and cells. In Pb3, C18:1 predominated in vesicles and C18:2 in cells. In Pb18, although C18:2 prevailed in both vesicles and cells, in vesicles it was proportionally much higher than other fatty acids. San Blas et al. [35] analyzed total fatty acids from *P. brasiliensis* Pb73, where palmitic acid was predominant, followed by linoleic, oleic, and stearic acid. In Pb141, Pb168, and Pb9, oleic acid was found in higher amounts, followed by linoleic and palmitic acid [36]. In isolate SN, linoleic acid was the predominant fatty acid, followed by palmitoleic acid and palmitic acid [39]. All these discrepancies, however, might reflect differences in the isolate source and general culture conditions. It is well established that significant differences in fatty acid composition can occur upon variations in culture temperature, age, and medium among intraspecific strains of microorganisms [40]–[42]. It remains to be tested whether the variations described in the present work are intrinsic to each isolate, if they represent variations characteristic of different phylogenetic groups, or if they are related to growth conditions.

Although ergosterol has been considered the characteristic sterol of fungal membranes, it is currently known that other types can prevail in some species [43]. In our present analysis, brassicasterol was predominant in extracellular vesicles and *P. brasiliensis* cells, followed by ergosterol and lanosterol, whereas in Pb18 and Pb3 cell wall preparations only brassicasterol was identified (Longo, Nakayasu, Almeida, and Puccia, unpublished). These results are similar to those described for Pb73 yeast cells [35], where brassicasterol was the most abundant sterol followed by lanosterol, and ergosterol; in the mycelium phase, ergosterol predominated over brassicasterol. Recently, the sterol biosynthetic pathway was characterized in *Paracoccidioides*, where lanosterol and brassicasterol are precursors, whereas ergosterol is the end product [44]. In *C. neoformans* extracellular vesicles, ergosterol and an obtusifoliol analog were the only sterols found [2].

We detected two species of neutral GSLs in *P. brasiliensis* extracellular vesicles, namely, Hex-C18:0-OH/d19:2-Cer and Hex-C18:1-OH/d19:2-Cer. Our results are consistent with those from Toledo et al. [14] for total GSL from Pb18 and based on their results we are assuming that the CMH here identified in vesicles is a glucosylceramide (GlcCer), although we have not determined the hexose nature. On the other hand, galactose can alternatively be found in fungal CMH, however less often [45]. GlcCer was associated with *P. brasiliensis* phase transition, since the proportion of (E)-A^3^-unsaturated fatty acid, which is only seen in fungal GlcCer, shifted from 50% in mycelium to only 15% in yeasts [14]. A similar difference in abundance identified in yeast cells was presently seen in *P. brasiliensis* extracellular vesicles, considering that the most abundant species at *m/z* 876.8 corresponds to Hex-Cer with saturated fatty acid, and the minor ion species at *m/z* 874.7 corresponds to Hex-Cer with (E)-A^3^-unsaturated fatty acid. GlcCer seems to be vital to normal *Aspergillus fumigatus* development, since inhibitors of ceramide glycosylation evoked impaired hyphal growth, spore germination and normal cell cycle progression [46]. In *C. albicans*, mutants lacking GlcCer retained normal growth and the capacity to form hyphae [47]: however its virulence was impaired [48]. CMH has previously been detected in *C. neoformans* extracellular vesicles [2] and cell wall, where it participates in cell budding and fungal growth [49]. It constitutes an important virulence factor in *C. neoformans*, since knockout mutants for GlcCer synthase activity were avirulent in an intranasal CBA/J mouse model of cryptococcosis [18]. In this opportunistic yeast, GlcCer seems to be necessary for growth in neutral pH environments.

Released vesicles need to transpose the cell wall in order to reach the extracellular milieu, as shown in *C. neoformans* [2]. Part of the vesicles might stay on the surface and enroll in different functions. That would add constituents to the cell wall composition, including membrane lipids. We have recently analyzed the lipid composition of isolated cell wall preparations from Pb18 and Pb3 and in general the results were similar to those described here for extracellular vesicles (Longo, Nakayasu, Almeida, and Puccia, unpublished).

Lipid composition could be a key element to help elucidate the biogenesis of extracellular vesicles. Thus far, lipids typically found in detergent-resistant membrane domains such as cholesterol, sphingomyelin, and glycolipids, such as ganglioside GM3, were also enriched in exosomes [26], suggesting an important role for these microdomains in vesicle biogenesis. In our analysis, we found that *P. brasiliensis* extracellular vesicles seem to be enriched in sterols and glycolipids, but we failed to find sphingomyelin or other phosphosphingolipids. However, sphingomyelin is apparently a minor component in *P. brasiliensis* [35]. The fact that extracellular vesicles have different sterol and fatty acid composition compared to whole yeast cells would support the idea that vesicles are derived from specific organelles. In this sense, a detailed lipidomic analysis of isolated organelles would bring more insights into the fungal vesicle biogenesis, which seems to rely to both conventional and unconventional pathways of secretion [8], [9].

In sum, our data help to understand the composition of fungal extracellular vesicles and will hopefully be useful to study their biogenesis and role in host-pathogen interactions and immunomodulation.

## Supporting Information

Figure S1 **Tandem-MS spectrum of a major phosphatidylcholine (PC) species (C18:2/C18:1-PC).** Samples were dissolved in methanol, containing 10 mM LiOH, and analyzed by ESI-MS/MS in the positive-ion mode. Fragmentation was carried out by total-ion mapping using pulsed-Q dissociation (PQD), and spectra were annotated manually. Cho, choline; Me_3_N, trimethylamine; P, phosphate. *m/z,* mass to charge ratio.
(TIF)

Figure S2 **Tandem-MS spectrum of a major phosphatidylethanolamine (PE) species (C18:2/C18:2-PE).** Samples were dissolved in methanol, containing 0.05% formic acid (FA), and 0.05% NH_4_OH, and analyzed by ESI-MS/MS in the negative-ion mode. Fragmentation was performed by total-ion mapping using pulsed-Q dissociation (PQD), and spectra were annotated manually. GroPEtN, glycerophosphoethanolamine.
(TIF)

Figure S3 **Tandem-MS spectrum of a major phosphatidylcholine (PC) species (C16:0/C18:2-PC).** Samples were dissolved in methanol, containing 0.05% FA and 0.05% NH_4_OH, and analyzed by ESI-MS/MS in the negative-ion mode. Fragmentation was carried out by total-ion mapping using pulsed-Q dissociation (PQD), and spectra were annotated manually. GroP, glycerophosphate; MeCl, methylchloride.
(TIF)

Figure S4 **Tandem-MS spectrum of a major phosphatidylserine (PS) species (C16:0/C18:2-PS).** Samples were dissolved in methanol, containing 0.05% FA, 0.05% NH_4_OH, and analyzed by ESI-MS/MS in the negative-ion mode. Fragmentation was carried out by total-ion mapping using pulsed-Q dissociation (PQD), and spectra were annotated manually. GroP, glycerophosphate; Ser, serine.
(TIF)

Figure S5 **Tandem-MS spectrum of a major phosphatidylinositol (PI) species (C16:0/C18:2-PI).** Samples were dissolved in methanol, containing 0.05% FA, 0.05% NH_4_OH, and analyzed by ESI-MS/MS ion in the negative-ion mode. Fragmentation was performed by total-ion mapping using pulsed-Q dissociation (PQD), and spectra were annotated manually. GroP, glycerophosphate; InsP, phosphoinositol.
(TIF)

Figure S6 **Tandem-MS spectrum of a major phosphatidylglycerol (PG) species (C16:0/C18:1-PG).** Samples were dissolved in methanol, containing 0.05% FA, 0.05% NH_4_OH, and analyzed by ESI-MS/MS in the negative-ion mode. Fragmentation was carried out by total-ion mapping using pulsed-Q dissociation (PQD), and spectra were annotated manually. GroP, glycerophosphate.
(TIF)

Figure S7 **Tandem-MS spectrum of a major phosphatidic acid (PA) species (C18:2/C18:1-PA).** Samples were dissolved in methanol, containing 0.05% FA, 0.05% NH_4_OH, and analyzed by ESI-MS/MS in the negative-ion mode. Fragmentation was carried out by total-ion mapping using pulsed-Q dissociation (PQD), and spectra were annotated manually. GroP, glycerophosphate.
(TIF)
