# Correction: Lipidomic Analysis of Extracellular Vesicles from the Pathogenic Phase of *Paracoccidioides brasiliensis*

**DOI:** 10.1371/annotation/08ed7ef4-7f80-4aed-9929-98d39c3ca83f

**Published:** 2012-10-12

**Authors:** Milene C. Vallejo, Ernesto S. Nakayasu, Larissa V. G. Longo, Luciane Ganiko, Felipe G. Lopes, Alisson L. Matsuo, Igor C. Almeida, Rosana Puccia

There are errors in Table 1. A correct version of Table 1 can be seen here: 

**Figure:**
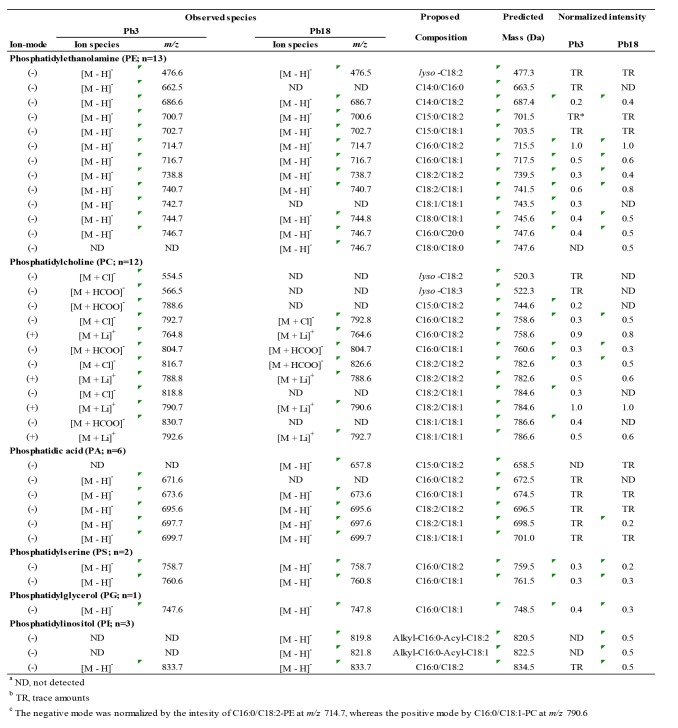


 [^] 

