# Supplementary figures and images for: Lipidomic Analysis of Extracellular Vesicles from the Pathogenic Phase of Paracoccidioides brasiliensis
Source: PLoS One. 2012 Jun 22;7(6):e39463. doi: 10.1371/journal.pone.0039463 (PMC3382159; doi:10.1371/journal.pone.0039463)

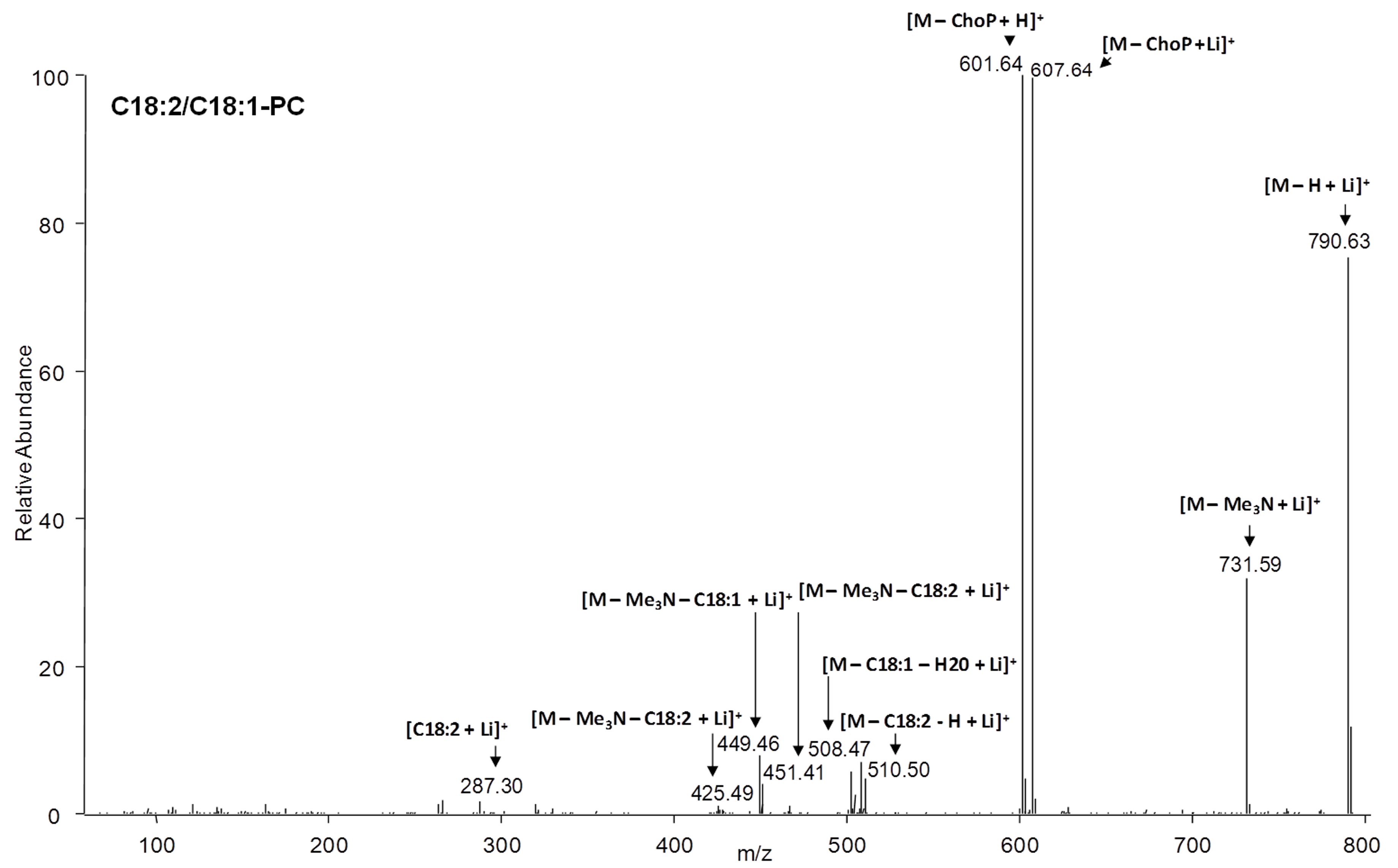

Supplement: Figure S1 — Tandem-MS spectrum of a major phosphatidylcholine (PC) species (C18:2/C18:1-PC). Samples were dissolved in methanol, containing 10 mM LiOH, and analyzed by ESI-MS/MS in the positive-ion mode. Fragmentation was carried out by total-ion mapping using pulsed-Q dissociation (PQD), and spectra were annotated manually. Cho, choline; Me3N, trimethylamine; P, phosphate. m/z, mass to charge ratio. (TIF) [file pone.0039463.s001.tif]

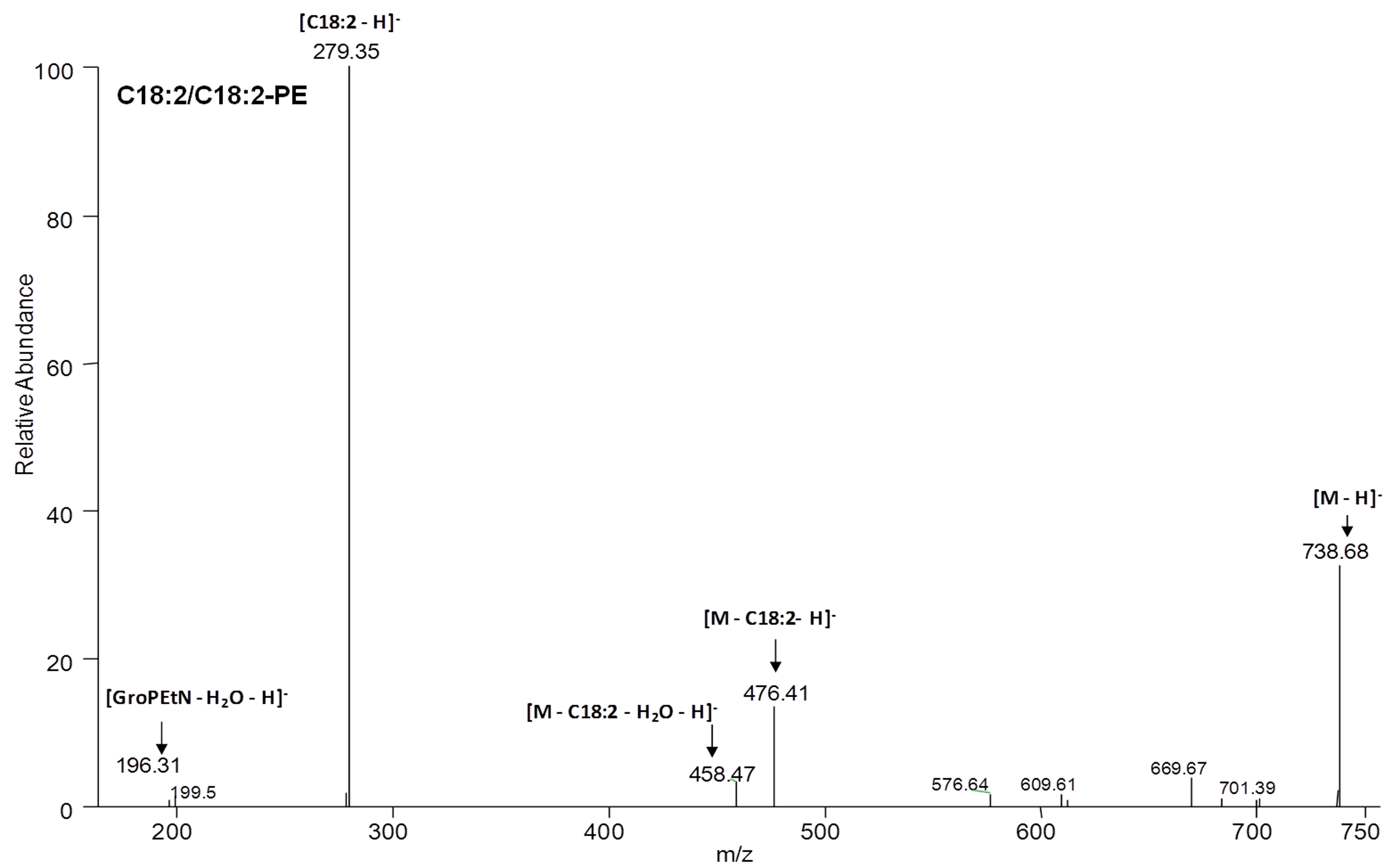

Supplement: Figure S2 — Tandem-MS spectrum of a major phosphatidylethanolamine (PE) species (C18:2/C18:2-PE). Samples were dissolved in methanol, containing 0.05% formic acid (FA), and 0.05% NH4OH, and analyzed by ESI-MS/MS in the negative-ion mode. Fragmentation was performed by total-ion mapping using pulsed-Q dissociation (PQD), and spectra were annotated manually. GroPEtN, glycerophosphoethanolamine. (TIF) [file pone.0039463.s002.tif]

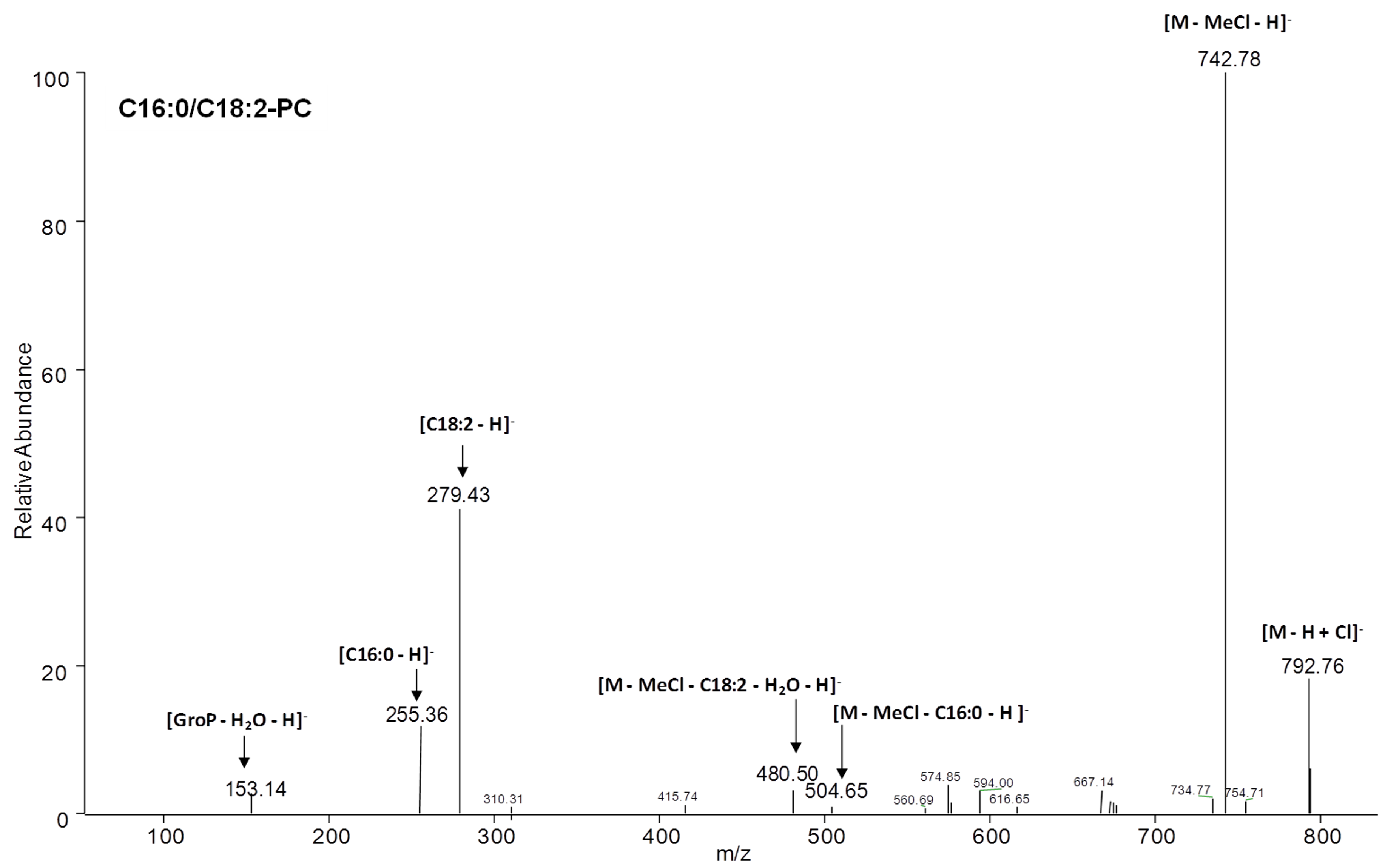

Supplement: Figure S3 — Tandem-MS spectrum of a major phosphatidylcholine (PC) species (C16:0/C18:2-PC). Samples were dissolved in methanol, containing 0.05% FA and 0.05% NH4OH, and analyzed by ESI-MS/MS in the negative-ion mode. Fragmentation was carried out by total-ion mapping using pulsed-Q dissociation (PQD), and spectra were annotated manually. GroP, glycerophosphate; MeCl, methylchloride. (TIF) [file pone.0039463.s003.tif]

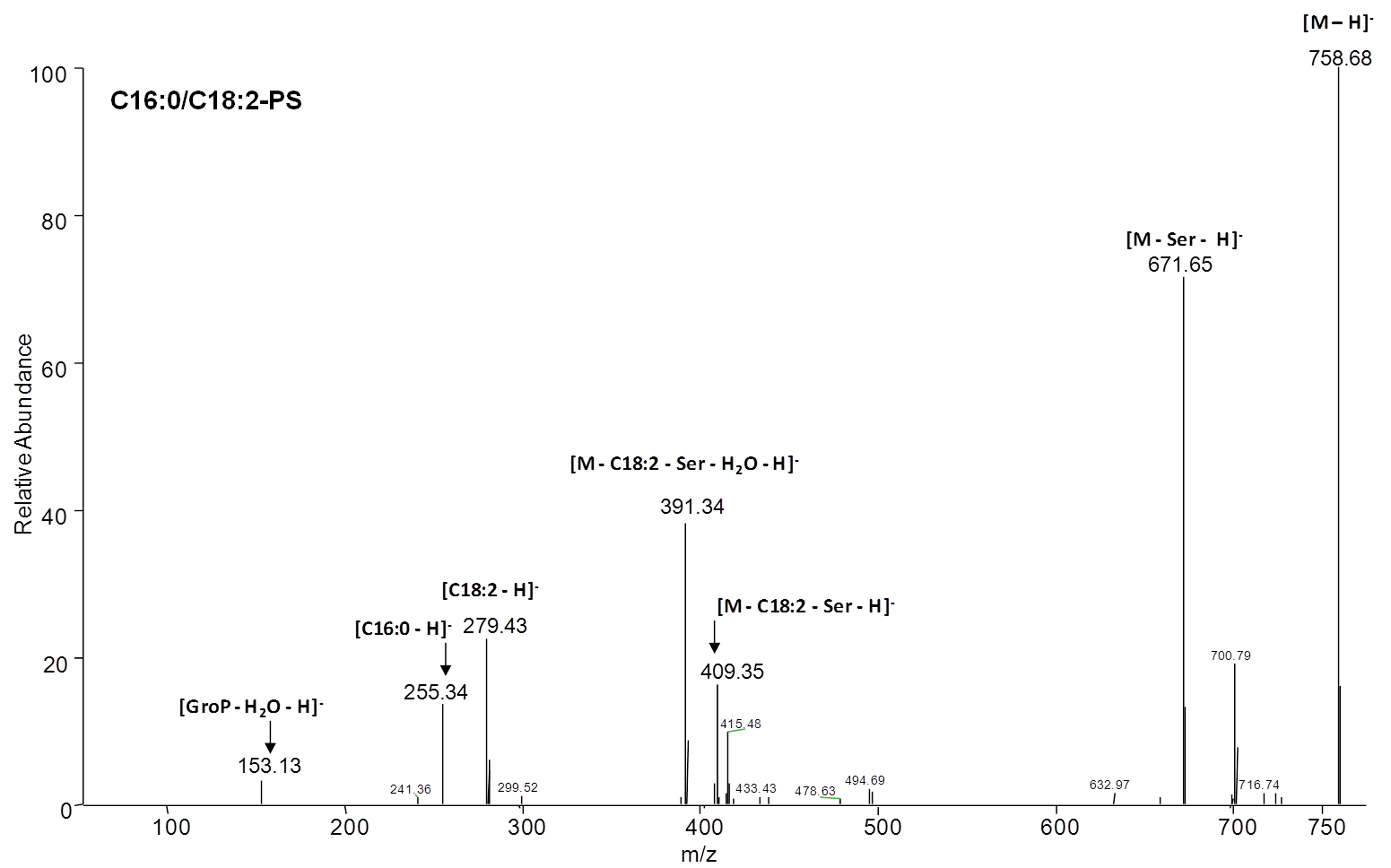

Supplement: Figure S4 — Tandem-MS spectrum of a major phosphatidylserine (PS) species (C16:0/C18:2-PS). Samples were dissolved in methanol, containing 0.05% FA, 0.05% NH4OH, and analyzed by ESI-MS/MS in the negative-ion mode. Fragmentation was carried out by total-ion mapping using pulsed-Q dissociation (PQD), and spectra were annotated manually. GroP, glycerophosphate; Ser, serine. (TIF) [file pone.0039463.s004.tif]

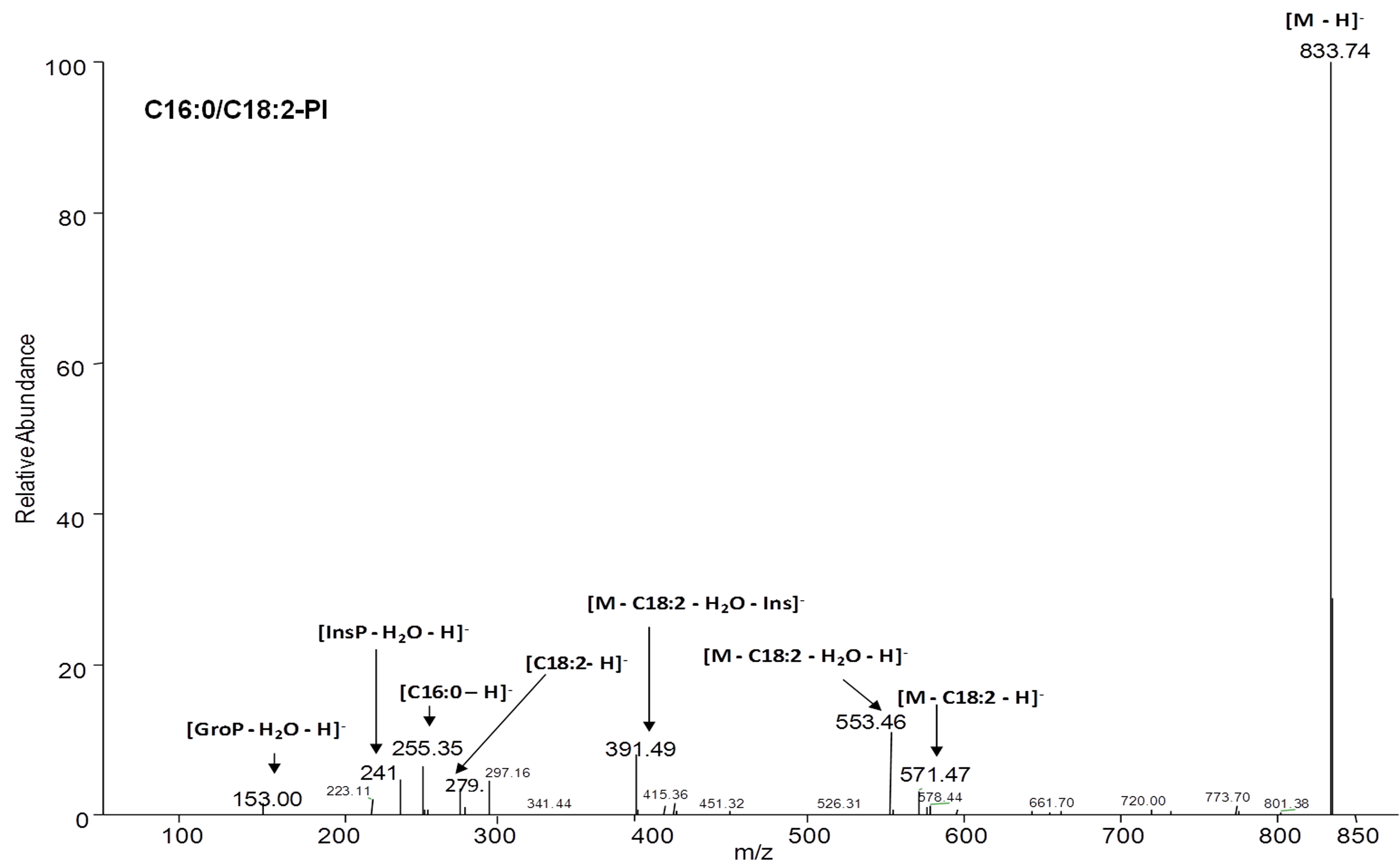

Supplement: Figure S5 — Tandem-MS spectrum of a major phosphatidylinositol (PI) species (C16:0/C18:2-PI). Samples were dissolved in methanol, containing 0.05% FA, 0.05% NH4OH, and analyzed by ESI-MS/MS ion in the negative-ion mode. Fragmentation was performed by total-ion mapping using pulsed-Q dissociation (PQD), and spectra were annotated manually. GroP, glycerophosphate; InsP, phosphoinositol. (TIF) [file pone.0039463.s005.tif]

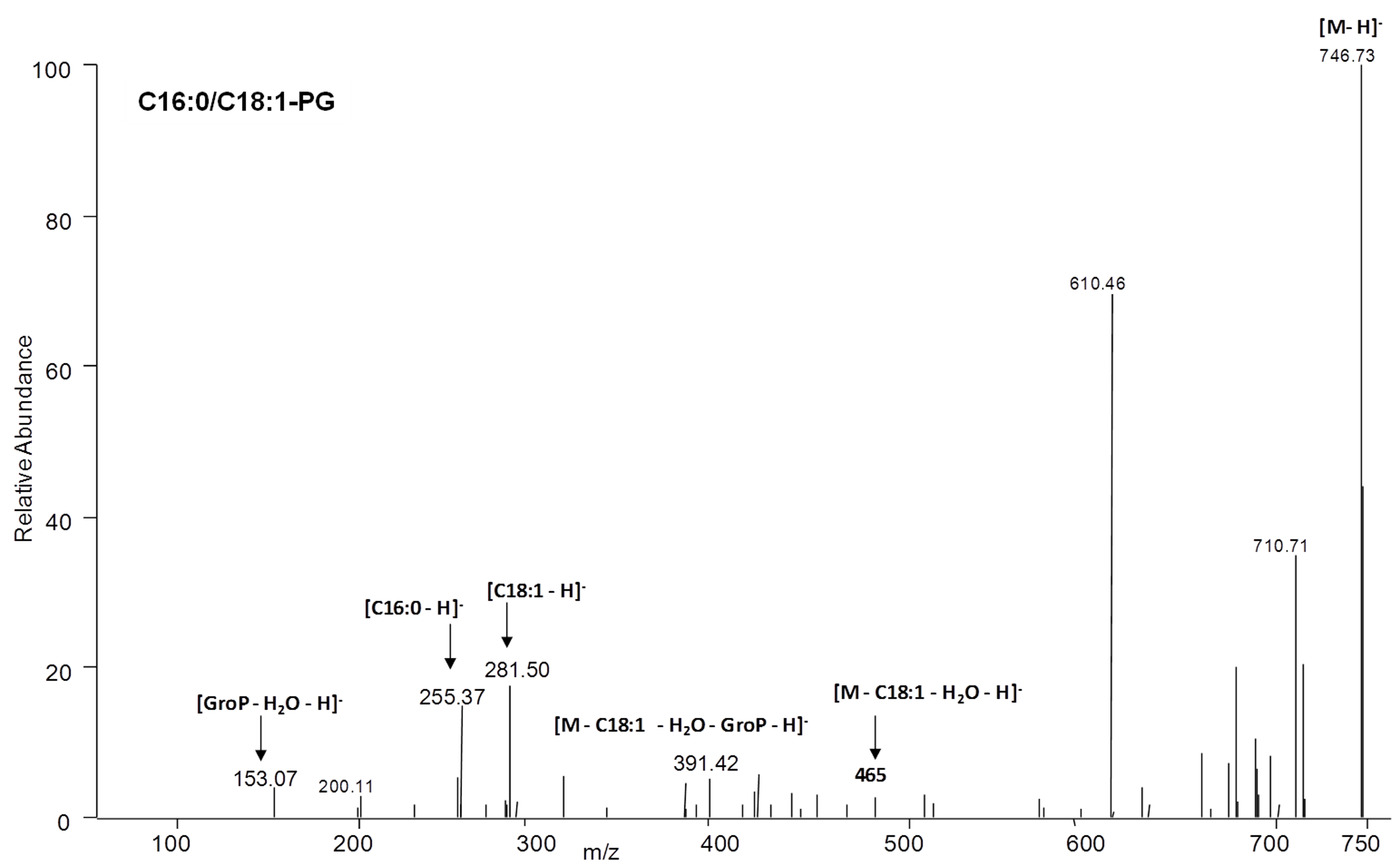

Supplement: Figure S6 — Tandem-MS spectrum of a major phosphatidylglycerol (PG) species (C16:0/C18:1-PG). Samples were dissolved in methanol, containing 0.05% FA, 0.05% NH4OH, and analyzed by ESI-MS/MS in the negative-ion mode. Fragmentation was carried out by total-ion mapping using pulsed-Q dissociation (PQD), and spectra were annotated manually. GroP, glycerophosphate. (TIF) [file pone.0039463.s006.tif]

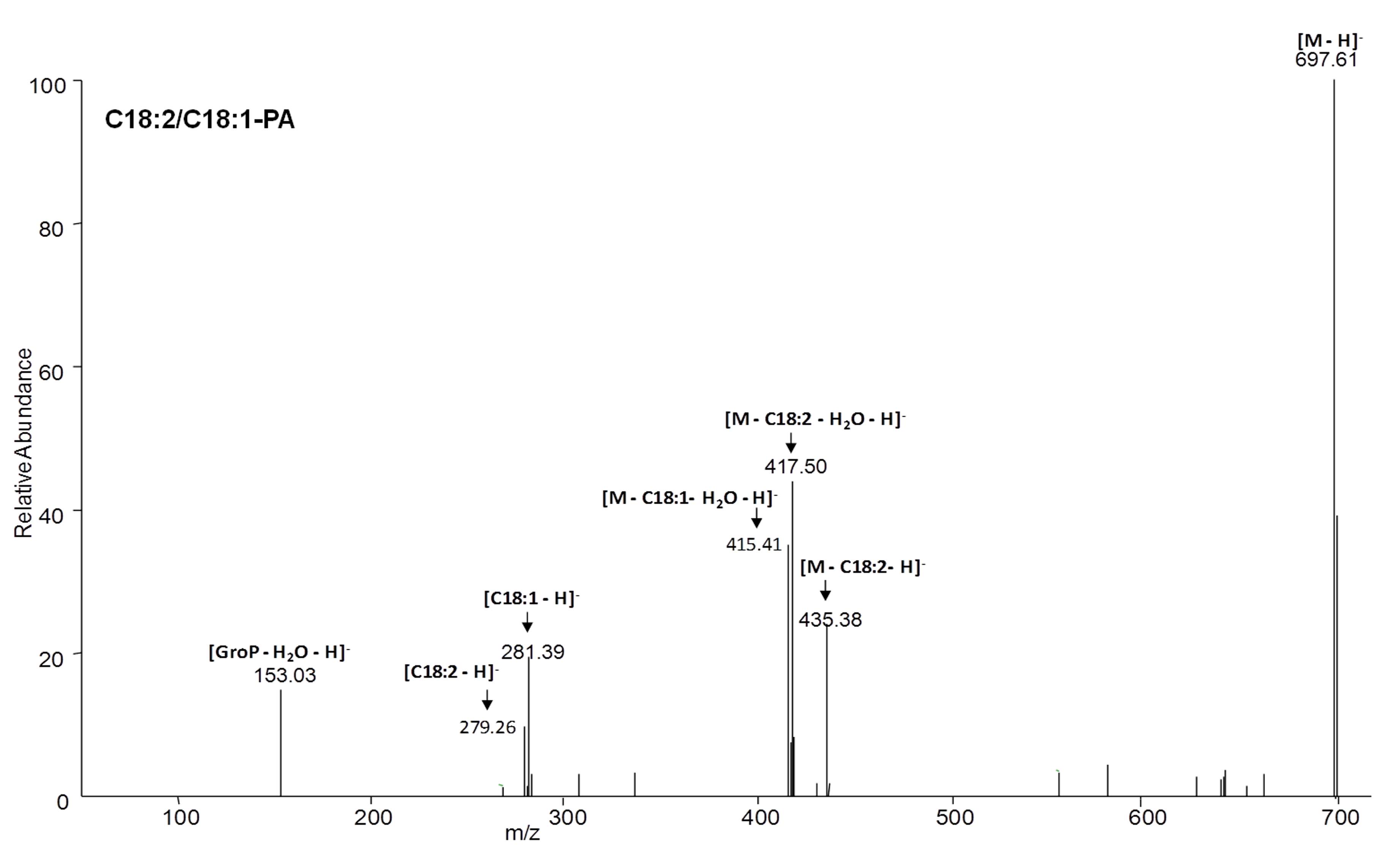

Supplement: Figure S7 — Tandem-MS spectrum of a major phosphatidic acid (PA) species (C18:2/C18:1-PA). Samples were dissolved in methanol, containing 0.05% FA, 0.05% NH4OH, and analyzed by ESI-MS/MS in the negative-ion mode. Fragmentation was carried out by total-ion mapping using pulsed-Q dissociation (PQD), and spectra were annotated manually. GroP, glycerophosphate. (TIF) [file pone.0039463.s007.tif]
